# *Leishmania major* Glycosylation Mutants Require Phosphoglycans (*lpg2* ^−^) but Not Lipophosphoglycan (*lpg1* ^−^) for Survival in Permissive Sand Fly Vectors

**DOI:** 10.1371/journal.pntd.0000580

**Published:** 2010-01-12

**Authors:** Anna Svárovská, Thomas H. Ant, Veronika Seblová, Lucie Jecná, Stephen M. Beverley, Petr Volf

**Affiliations:** 1 Department of Parasitology, Faculty of Science, Charles University in Prague, Czech Republic; 2 Keele University, Keele, Staffordshire, United Kingdom; 3 Department of Molecular Microbiology, Washington University School of Medicine, St. Louis, Missouri, United States of America; Instituto Oswaldo Cruz, Fiocruz, Brazil

## Abstract

**Background:**

Sand fly species able to support the survival of the protozoan parasite *Leishmania* have been classified as permissive or specific, based upon their ability to support a wide or limited range of strains and/or species. Studies of a limited number of fly/parasite species combinations have implicated parasite surface molecules in this process and here we provide further evidence in support of this proposal. We investigated the role of lipophosphoglycan (LPG) and other phosphoglycans (PGs) in sand fly survival, using *Leishmania major* mutants deficient in LPG (*lpg1* ^−^), and the phosphoglycan (PG)-deficient mutant *lpg2* ^−^. The sand fly species used were the permissive species *Phlebotomus perniciosus* and *P. argentipes*, and the specific vector *P. duboscqi*, a species resistant to *L. infantum* development.

**Principal Findings:**

The *lpg2* ^−^ mutants did not survive well in any of the three sand fly species, suggesting that phosphoglycans and/or other *LPG2*-dependent molecules are required for parasite development. *In vitro*, all three *L. major* lines were equally resistant to proteolytic activity of bovine trypsin, suggesting that sand fly-specific hydrolytic proteases or other factors are the reason for the early *lpg2* ^−^ parasite killing. The *lpg1* ^−^ mutants developed late-stage infections in two permissive species, *P. perniciosus* and *P. argentipes*, where their infection rates and intensities of infections were comparable to the wild type (WT) parasites. In contrast, in *P. duboscqi* the *lpg1* ^−^ mutants developed significantly worse than the WT parasites.

**Conclusions:**

In combination with previous studies, the data establish clearly that LPG is not required for *Leishmania* survival in permissive species *P. perniciosus* and *P. argentipes* but plays an important role in the specific vector *P. duboscqi*. With regard to PGs other than LPG, the data prove the importance of *LPG2*-related molecules for survival of *L. major* in the three sand fly species tested.

## Author Summary

Phlebotomine sand flies are small blood-feeding insects, medically important as vectors of protozoan parasites of the genus *Leishmania*. Sand flies species can be divided roughly into two groups, termed specific or permissive, depending on their ability to support development of one or a few strains vs. a broad spectrum of these parasites. In this study, we explored the ability of two *Leishmania major* glycocalyx mutants to survive within these different types of vectors. The *lpg1* ^−^ mutant, which specifically lacks lipophosphoglycan (LPG), was able to survive normally in two permissive species, *Phlebotomus argentipes* and *P. perniciosus*, but was only able to survive within the specific species *P. duboscqi* for a limited time prior to dissolution of the peritrophic matrix. Consistent with its classification as a specific sand fly vector, *P. duboscqi* was not able to support development of *L. infantum*. The *lpg2* ^−^ *L. major* mutant, which is a broader mutant and lacks all phosphoglycans including LPG and proteophosphoglycans, was unable to survive in all the three vector species tested. This study extends the knowledge on the role of *Leishmania major* surface glycoconjugates to development in three important vector species and gives supporting evidence for the existence of an LPG-independent mechanism for survival in sand flies, as well as the importance of *LPG2*-dependent glycoconjugates in parasite survival.

## Introduction

The distribution of diseases caused by the protozoan parasite *Leishmania* is limited by the distribution of the sand fly vectors and their capacity to support parasite development. Survival of *Leishmania* parasites during bloodmeal digestion and their attachment to the midgut epithelium have been identified as two critical steps determining the vector competence. Based upon experimental tests of their ability to support development of wide or limited range of *Leishmania* species, sand flies have been classified as permissive or specific vectors [1]. According to previous investigations, there is a close evolutionary fit between *Phlebotomus papatasi* and *P. sergenti* with *Leishmania major* and *L. tropica* respectively, as other *Leishmania* species survive poorly in these sand fly hosts. In contrast, other sand flies tested (*P. argentipes*, *P. halepensis*, *P. arabicus* and *Lutzomyia longipalpis*) were broadly permissive to the development of different *Leishmania* parasites. This classification is based on experimental studies and does not imply the constraints of natural transmissions (vector capacity). However, it reflects the vector competence of permissive sand flies for transmission of various parasites (for review see [1],[2]).

*Leishmania* surface molecules have been strongly implicated in parasites survival within sand fly vectors. *Leishmania* promastigotes synthesise an abundance of glycoconjugates composed of polymeric units based upon a conserved Gal-Man-P phosphoglycan (PG) repeating unit for review see [3]. These include the membrane-attached glycosylphosphatidyl-inositol (GPI) anchored liposphophoglycan (LPG) and proteophosphoglycan (PPG), as well as secreted forms of PPGs and secretory acid phosphatases (sAPs). PGs have been implicated in the early survival of *L. donovani* within the bloodfed midgut [4], presumably by conferring resistance to, or by modulating the activity of digestive enzymes. The role of these molecules in sand fly interactions has been studied by biochemical methods using purified LPG, PPG and other molecules, although the shared PG determinants across molecules makes the assignment of function to specific PG classes problematic [5],[6]

Thus, complementary to studies using purified PGs, researchers have generated and studied the behaviour of mutants lacking specific LPG or PG biosynthetic genes, which in turn affect the synthesis of subsets of PG-bearing molecules. The *lpg1* ^−^ mutant lacks the *LPG1* gene (GenBank accession no. AF234766) which encodes a galactofuranosyltransferase required for synthesis of the LPG glycan core, rendering such mutants specifically deficient in LPG alone [7]. In contrast, the *Leishmania major lpg2* ^−^ mutant lacks the *LPG2* gene (GenBank accession no. AF350492) encoding a Golgi GDP-sugar transporter. As a result, these parasites fail to synthesize LPG and all other PGs [8],[9]. Recent studies suggest that the *lpg2* ^−^ mutant may lack additional, as yet unidentified and likely rare glycoconjugates [10]. Importantly, the LPG and virulence phenotypes of the *L. major lpg1* ^−^ and *lpg2* ^−^ lines in both mammalian and sand fly infections were restored to WT following re-expression of the cognate gene, confirming the genetic specificity of the virulence defects [4], [5], [7], [9]–[13].

The genetic and biochemical approaches above have established that LPG, the dominant surface glycoconjugate of *Leishmania* promastigotes, mediates attachment to the midgut epithelium in *P. papatasi*, preventing the loss *L. major* parasites during blood meal excretion, as *lpg1* ^−^ parasites survive the initial stages of fly infection but are subsequently lost due to a failure to bind to a sand fly midgut LPG receptor [4],[14],[15]. Notably *P. papatasi* is considered to be a highly specific vector, in that other species of *Leishmania* are unable to establish infection in this sand fly [16]–[19]. In contrast, in *P. arabicus* and *Lu. longipalpis*, two species shown to be permissive vectors by virtue of their ability support development of various *Leishmania* species, LPG-deficient *lpg1* ^−^ *L. major* develop and produce mature infections [20]. From these data Myskova *et. al* hypothesised that LPG is required in specific vectors, while in permissive vectors *Leishmania* bind via an LPG independent mechanism.

In this work we further test this hypothesis by infections of three additional sand fly species with WT and mutant *L. major*. In addition, we attempted to assess the importance of LPG and other *LPG2*-dependent molecules in protection against proteolytic attack by exposing the mutant parasite lines to the action of bovine trypsin *in vitro*. Importantly, the three sand fly species used in the study are important vectors known to transmit *Leishmania*. *Phlebotomus duboscqi* is a vector of cutaneous leishmaniasis caused by *L. major* in sub-Saharan Africa [21],[22]. It is a sister species of *P. papatasi* and belongs to the same subgenus. Unlike *P. papatasi*, some populations of *P. duboscqi* have been shown experimentally to support development of *L. tropica* [16]. Midgut glycosylation and the degree of permissivity of this species are unclear. We addressed the question of permissivity of *P. duboscqi* sand flies in this study by infecting them with *L. infantum*. The other two species used are permissive vectors transmitting parasites of *L. donovani* complex. Myskova *et al.* [20] demonstrated that both, *P. argentipes* and *P. perniciosus* posses midgut glycoproteins with HPA (*Helix pomatia* agglutinin, lectin with specificity to N-acetyl-D-galactosamine)-binding epitopes. *Phlebotomus argentipes* is a vector of visceral anthroponotic leishmaniasis caused by *Leishmania donovani* in the Indian subcontinent [23]. In experimental conditions it supports development of *L. donovani*, *L. amazonensis*, *L. major* and *L. tropica* [4],[19],[24]. *Phlebotomus perniciosus* is a vector of *Leishmania infantum* in the western Mediterranean and in experimental conditions it supports the development of *L. tropica* (V.S. and P.V., unpublished results).

## Materials and Methods

### Parasites

*Leishmania infantum* MHOM/TR/2000/OG-VL and three lines of *Leishmania major* LV39 clone 5 (MRHO/SU/1959/Neal P) [25] were used in this work. The *L. major LPG1* and *LPG2* knockout mutants *lpg1* ^−^ and *lpg2* ^−^ were generated in the LV39 clone 5 background previously [7],[9]. Parasites were maintained at 23°C on medium 199 supplemented with 20% foetal calf serum (Gibco) and gentamicin (50µg/ml). For the mutated lines, selection antibiotics were added to the culture medium as follows: hygromycin B (15µg/ml) for the *lpg2* ^−^ mutant; hygromycin (15 µg/ml) and puromycin (11µg/ml) for the *lpg1* ^−^ mutant. Prior to sand fly infections, parasites were washed by centrifugation and resuspended in saline solution.

### Sand fly colonies

Laboratory colonies of three sand fly species were used: *Phlebotomus perniciosus* (originally from Spain), *P. argentipes* (originally from India) and *P. duboscqi* (originally from Senegal). Colonies were maintained in conditions described previously [26]. Adults were maintained at 26°C and fed on 50% sucrose *ad libitum*.

### *Leishmania* development in sand flies

Female sand flies (5–10 days old) were fed through a chick skin membrane with 4–5 day old promastigotes at cell density of 5×10^6^ (*P. duboscqi* infections with *L. major*) or 1×10^6^ promastigotes/ml (all other infections, including *P. duboscqi* with *L. infantum*) in heat inactivated rabbit blood (Bioveta, Ivanovice). Blood-engorged females were maintained at 26°C with access to cotton wool soaked in 50% solution of sugar in distilled water and sacrificed for microscopical examination and counting of parasites in the midgut 2 and 5 or 9 days post infection. Intensity of infection was graded as light (<100 parasites/gut), moderate (100–1000 parasites/gut) or heavy (>1000 parasites/gut) as described previously [27]. *Phlebotomus perniciosus* and *P. duboscqi* defecate between 75 and 95 hours post-feeding [28] and *L. major* colonized their stomodeal valve on days 7–9 post-feeding [27]. Preliminary experiments showed that *Phlebotomus argentipes* defecates 2–3 days post-feeding and parasites reached the stomodeal valve by day 5 already.

Experiments were repeated twice. The χ^2^ test was used for comparison of infection rates (number of infected versus uninfected females) and intensities of infection (heavy, moderate light, zero) between the WT and the mutant lines using S-PLUS 2000 programme.

### Parasite susceptibility to bovine trypsin

Promastigotes of a 4-day culture were washed in medium 199 (M 199), adjusted to the concentration of 3×10^6^ cells/ml of M 199 and exposed to bovine trypsin (13.500 BAEE units/ml) (Sigma) alone or bovine trypsin plus 6% human haemoglobin (Sigma; one BAEE unit will produce a _Δ_A^253^ of 0.001 per min at pH 7.6 at 25 °C using benzoyl-L-arginine ethyl ester (BAEE) as substrate). In control groups, parasites were cultivated in M 199 alone. After 24 hours at 23°C, parasite numbers were determined by haemocytometer counting. Assay was performed in triplicate and the experiment was repeated twice. Data were evaluated statistically by means of ANOVA test.

### Detection of glycoconjugates in *P. duboscqi* midgut lysates

Midguts of female *P. duboscqi* were homogenized in Tris buffer (20mM Tris, 150mM NaCl, pH 7.6) and proteins were analyzed by SDS PAGE (10% gel, reducing conditions, 10 µg protein per lane) followed by western blotting. The nitrocellulose membrane was incubated in Tris buffer with 0.05% Tween 20 (Tris-Tw) with 5% bovine serum albumin overnight and then with biotinylated lectins (Sigma) in Tris-Tw with 1% BSA in the following concentrations: concanavalin A (Con, 2.5 µg/ml), *Pisum sativum* agglutinin (PSA, 2.5 µg/ml), *Helix pomatia* agglutinin (HPA, 1 µg/ml), *Ricinus communis* agglutinin (RCA, 0.5 µg/ml), Soybean agglutinin (SBA, 10 µg/ml). After repeated washing the blots were incubated with streptavidin peroxidase (2.5 µg/ml) in Tris-Tw and developed in 3,3′-diaminobenzidine solution. The specificity of Con A and PSA reactions were controlled by addition of 250mM methyl-mannopyranoside as an inhibitory sugar.

## Results

### Development of *L. major* in *P. duboscqi*

**Figure 1:**
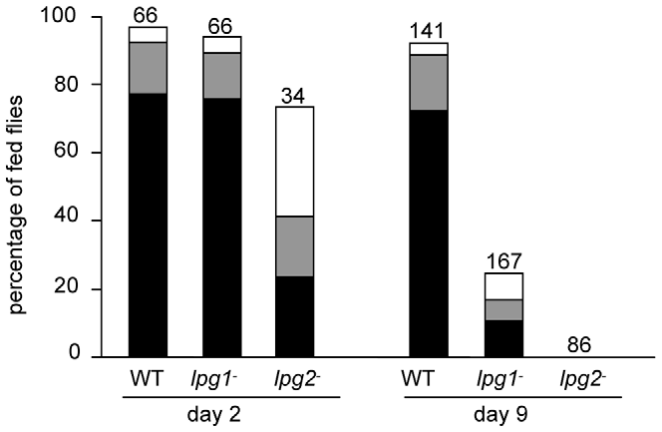
Development of *L. major* mutants in *P. duboscqi*. *Phlebotomus duboscqi* females were infected with *Leishmania major* wild type (WT) or mutants lacking LPG (*lpg1* ^−^) or all *LPG2*-dependent molecules (*lpg2* ^−^). Day 2 - dissection before defecation (48hours post-infection), day 9 - dissection after defecation. Infections were classified into three cathegories: heavy (more than 1000 promastigotes per gut) - black bars, moderate (100–1000) - grey bars, light (1–100) - white bars. Numbers above the bars indicate the number of dissected females.

*Phlebotomus duboscqi* sand flies were infected with WT, *lpg1* ^−^ and *lpg2* ^−^ mutants of *L. major* in order to study the role of LPG and other PGs. On day 2 post-infection, no differences were observed between development of WT and the *lpg1* ^−^ line, with both showing very high rates of infection (97% and 93% respectively), with about 75% of heavy infections (Fig. 1). The *lpg2* ^−^ mutant survived less well however; their infection rate was significantly lower (74%; *P*<0.01), with only 24% of heavy infections.

In contrast to day 2, on day 9 post infection the *lpg1* ^−^ mutant survived much more poorly than WT, with only 25% of the flies retaining parasites, as opposed to 92% for WT (P<0.01). Moreover, the *lpg1* ^−^ line produced very few mature infections colonizing the stomodeal valve in only 10% of females. Notably the *lpg2* ^−^ line did not survive, as no *lpg2* ^−^ parasites were found in the midguts by day 9 (Fig. 1). Previous studies have established that phenotypes arising from the *lpg1* ^−^ and *lpg2* ^−^ mutants in diverse settings are specific, as they are reversed by complementation with the cognate gene, and thus do not arise as a result of nonspecific culture passage or other sources [4],[7],[9],[13]. Thus these mutant data argue that, as seen previously in the specific sand fly *P. papatasi*, LPG is required for late but not early survival [4], while *LPG2* is important for early survival and essential for late stage survival.

### Development of *L. major* lines in *P. argentipes*

**Figure 2:**
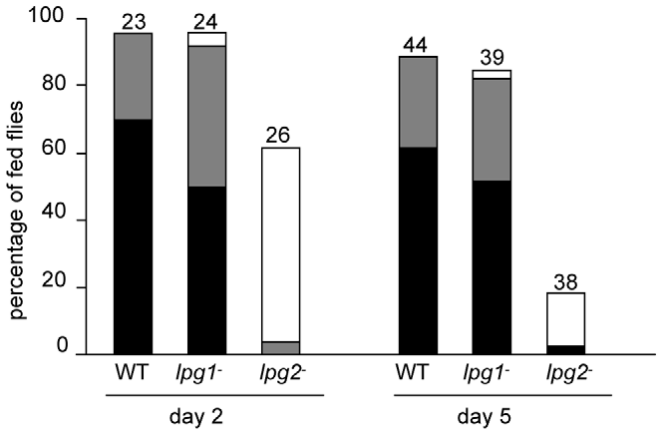
Development of *L. major* mutants in *P. argentipes*. *Leishmania major* lines tested were the same as in Fig. 1. Day 2 - dissection before defecation (48 hours post-infection); day 5 - dissection after defecation. The intensity of infections was evaluated as described in Fig. 1. Numbers above the bars indicate the number of dissected *P. argentipes* females.

Similar to the results obtained in *P. duboscqi*, on day 2 no statistically significant differences were found between the WT and the *lpg1* ^−^ mutant, while the *lpg2* ^−^ mutant was severely impaired (**Fig. 2**). Very high infection rates (96%) were present in both the *WT* and *lpg1* ^−^ lines, with heavy infections in 70% and 50%, respectively. In contrast, *lpg2* ^−^ mutants were more severely affected, with infections seen in 62% of flies but with very low parasite loads (less than 100 *Leishmania*, except for 1 fly). The differences in infection intensity and rate between the *lpg2* ^−^ and the WT were statistically highly significant (*P*<0.01).

Day-5 dissections revealed a continuation of the trends described above during late stage infections. Females infected with WT and *lpg1* ^−^ mutants retained high infection rate and high parasite loads (no statistically significant differences between the lines). Also the localizations of parasites were remarkably similar; WT and *lpg1* ^−^ parasites reached the stomodeal valve in 46% and 48%, respectively. In contrast, *lpg2* ^−^ line showed a remarkable decrease in infection rate, with only 18% of positive females (highly significant difference from the WT parasites, *P*<0.01) and no colonization of the stomodeal valve.

### Development of *L. major* lines in *P. perniciosus*

On day 2, no significant differences were observed between the three lines, all of them survived well inside the peritrophic sac producing heavy infections in about 25% of females.

**Figure 3:**
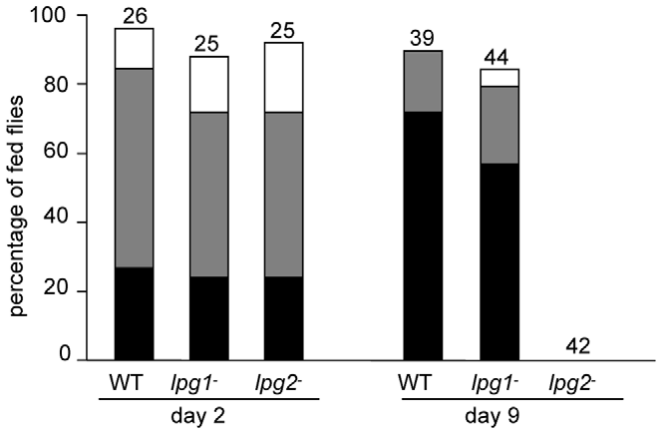
Development of *L. major* mutants in *P. perniciosus*. *Leishmania major* lines tested and evaluation of infections were the same as described in Fig. 1. Numbers above the bars indicate the number of dissected *P. perniciosus* females.

On day 9, *lpg2* ^−^ mutants were eliminated while *lpg1* ^−^ mutants developed similarly to the WT parasites (**Fig. 3**). WT and *lpg1* ^−^ lines developed mature infections colonizing the stomodeal valve with high parasite burdens in majority of females. In contrast, none of the *lpg2* ^−^ parasites were able to persist until day 9, suggesting that they were lost during defecation.

### *P. duboscqi* is refractory to *L. infantum*

In order to test the degree of permissivity of *P. duboscqi*, females of this species were infected with *L. infantum*, a parasite that is not transmitted by *P. duboscqi* in nature. Promastigotes were able to survive inside the peritrophic sac during digestion of the bloodmeal but they were not able to persist beyond defecation of the blood remnants. On day 2 post infective bloodmeal, 91% of the flies (11 of 12) were *Leishmania* positive while on day 8, no parasites were found in any female tested (n = 15) (data not shown). These results demonstrate that *P. duboscqi* is refractory to *L. infantum*.

### *P. duboscqi* midgut glycosylation

**Figure 4:**
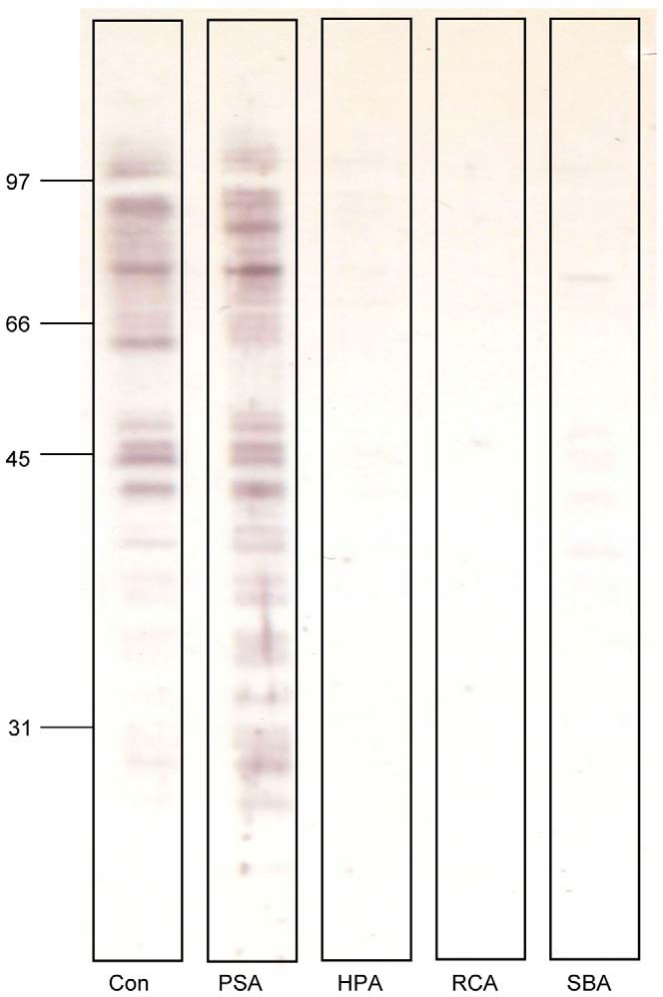
Western blot of *P. duboscqi* midgut proteins incubated with various lectins. Lectins concanavalin A (Con) and *Pisum sativum* agglutinin (PSA) specifically reacted with *P. duboscqi* midgut lysate. Reaction of lectins *Helix pomatia* agglutinin (HPA), *Ricinus communis* agglutinin (RCA) and Soybean agglutinin (SBA) were negative.

As detected by western blotting with lectins, *P. duboscqi* midgut lysate displays molecules that bind Con A and PSA, lectins detecting terminal mannose residues of glycans **(** **Fig. 4** **)**. Controls with inhibitory sugar (250mM methyl-mannopyranoside) confirmed the specificity of lectin reactions (data not shown). In contrast, HPA, RCA and SBA reactions were negative indicating absence of β-galactose or N-acetyl-D-galactosamine residues in the midgut glycoproteins **(** **Fig. 4** **)**. The lectin binding profile is similar to that previously observed in specific sand fly vectors *P. papatasi* and *P. sergenti*. In contrast, midgut lysates of all permissive sand fly species tested to date contain N-acetyl-D-galactosamine displaying glycoconjugates as detected by lectin affinity blotting [20].

### Effect of bovine trypsin on *Leishmania*

The *in-vitro* growth of *L. major* promastigotes of WT, *lpg1* ^−^ and *lpg2* ^−^ lines in M 199 medium was not affected either by bovine trypsin (13.500 BAEE units/ml) or by bovine trypsin plus 6% human hemoglobin.

No significant differences in numbers of viable parasites were observed between the groups in any of the three lines tested (P = 0.84).

## Discussion

The classic studies of Sacks and co-workers established a paradigm for the role of LPG in the survival of *L. major* and *L. donovani* in sand flies, mediated through binding of LPG to the sand fly midgut [4],[19]. In the case of *L. major* this interaction is now known to be mediated by the *P. papatasi* lectin PpGal ([15]). However in 2007 we reported the occurrence of LPG-independent sand fly survival, importantly only in sand fly species now termed ‘permissive’ as defined by their ability in experimental tests to support the development of a wide range of *Leishmania* species. In contrast, previous studies of LPG-dependency were now associated with ‘selective’ sand fly species (again defined by experimental tests as supporting the development of a narrow range of *Leishmania* species and/or isolates) ([20]). Given the implications of this new paradigm, it was important to garner additional data testing its validity by examining additional permissive and selective sand fly species, using the same well characterized LPG mutants studied previously. Additionally we have culled the literature and summarized the available data pertinent to this model (Table 1).

**Table 1:** Requirement for lipophosphoglycan (LPG) and other phosphoglycans (PGs) during *Leishmania* development in various sand fly species.

Vector species (colony origin)	Leishmania species	Interpretation	Mutant lines	Infection outcome	Reference
*P. papatasi* (Israel)	*L. major* (natural)	LPG required in late phase	*lpg1^−^* (LPG-deficient)	Low percentage of infected flies on day 5	Sacks *et al.* [4]
*P. papatasi* (Turkey)	*L. major* (natural)	LPG required in late phase	*lpg1^−^* (LPG-deficient)	Low percentage of infected flies on day 8	Myskova *et al.* [20]
*P. duboscqi* (Mali)	*L. major* (natural)	LPG required in late phase	*lpg1^−^* (LPG-deficient)	Fewer than a 1000 parasites/gut on day 7	Secundino *et al.*, (submitted)
		PGs required from early phase	*lpg2^−^* (PG-deficient)	Impaired on day 3; no parasites on day 7	
			*lpg5A^−^/5B^−^* (PG-deficient)	Impaired on day 3; low numbers of parasites on day 6	
*P. duboscqi* (Senegal)	*L. major* (natural)	LPG possibly required in late phase	*lpg1^−^* (LPG-deficient)	Comparable to WT on day 4, partially impaired on days 8 and 10	Boulanger *et al.* [32]
		*LPG2*-related molecules required from early phase	*lpg2^−^* (PG-deficient)	Impared on day 4, no parasites on days 8 and 10	
*P. duboscqi* (Senegal)	*L. major* (natural)	LPG required in late phase	*lpg1^−^* (LPG-deficient)	Comparable to *WT* on day 2; impaired on day 9	This study
		*LPG2*-related molecules required from early phase	*lpg2^−^* (PG-deficient)	Impaired on day 2; no parasites on day 9	
*Lu. longipalpis* (Brazil)	*L. major* (unnatural)	LPG-independent	*lpg1^−^* (LPG-deficient)	High level of infection on day 7	Myskova *et al.* [20]
			*lpg1^−^* (LPG-deficient)	High level of infection on day 7	Secundino *et al.*, (submitted)
		*LPG2*-related molecules required from early phase	*lpg2^−^* (PG-deficient)	Impaired on day 3; no parasites on day 7	Secundino *et al.*, (submitted)
	*L. mexicana* (unnatural)	LPG-independent	*lpg1^−^* (LPG-deficient)	Mature infections on day 7–9	Rogers *et al.* [29]
*P. arabicus* (Israel)	*L. major* (unnatural)	LPG-independent	*lpg1^−^* (LPG-deficient)	High level of infection on day 7	Myskova *et al.* [20]
*P. perniciosus* (Spain)	*L. major* (unnatural)	LPG-independent	*lpg1^−^* (LPG-deficient)	High level of infection on day 9	This study
		*LPG2*-related molecules required from early phase	*lpg2^−^* (PG-deficient)	Comparable to *WT* on day 2; no parasites on day 9	
*P. argentipes* (India)	*L. major* (unnatural)	LPG-independent	*lpg1^−^* (LPG deficient)	High level of infection on day 5	Thist study
		*LPG2*-related molecules required from early phase	*lpg2^−^* (PG-deficient)	Impaired on day 2; low numbers of parasites on day 5	
	*L. donovani* (natural)	LPG possibly required	R2D2 (LPG-deficient)	Severely impaired on day 5	Pimenta *et al.* [19]; Sacks *et al.* [4]
		PGs required from early phase	C3PO (PG-deficient)	Impaired on day 2; no parasites on day 5	Sacks *et al.* [4]
			*lpg2^−^* (PG-deficient)	Impaired on day 2; no parasites on day 5	Sacks *et al.* [4]

We found that *L. major* mutants specifically lacking LPG remain able to develop in the permissive vectors *P. perniciosus* and *P. argentipes* at levels resembling those of wild type parasites, with full midgut development and colonization of the stomodeal valve. These data suggest that in *P. perniciosus* and *P. argentipes* the LPG is required neither for parasite protection against digestive enzymes nor for midgut binding. LPG-independent development was previously reported for *L. major* in the permissive sand fly vectors *Lu. longipalpis* and *P. arabicus* [20], and *L. mexicana* development in *Lu. longipalpis* [29]. These data confirm and extend the results obtained in sand fly infections with all LPG-deficient *Leishmania* carried out by various laboratories to date (**Table 1**). Collectively these data provide strong support for the role of LPG in specific but not permissive sand fly vectors.

Within this data set we could only identify one potential exception, involving a study of the permissive vector *P. argentipes*, where the *L. donovani* LPG-deficient mutant line known as the ‘R2D2’, also defective in *LPG1* expression [30], did not survive when examined on day-5 post-infection [19]. Notably R2D2 was obtained following heavy mutagenesis and selection for LPG-deficiency, unlike the *lpg1* ^−^ *L. major* which was generated following precise gene targeting procedures [31]. It is well established in the genetics literature that mutagenesis frequently results in off-target deleterious effects. Our previous work established that the phenotypic alterations in the *lpg1* ^−^ and *lpg2* ^−^ arose specifically from alterations in these genes, as restoration of *LPG1* and *LPG2* function returned the phenotype to WT [7],[9]. In contrast, R2D2 failed this test, as restoration of *LPG1* expression to R2D2 only weakly restored both LPG and survival in *P. argentipes* [4].

In contrast to the permissive vectors, the development of *L. major lpg1* ^−^ mutants was severely impaired in the specific vector *P. duboscqi*. Although the early infections were similar to those of the WT parasites, there was a substantial decrease in the *lpg1* ^−^ infections rate after defecation of the bloodmeal. In very few females the *lpg1* ^−^ mutants produced mature late stage infections. Our results extend those reported in the study by Boulanger *et al.* [32] performed with a small number of sand flies. Similar results with *L. major lpg1* ^−^ mutants in *P. duboscqi* were recently obtained by Secundino *et al.* (submitted) (**Table 1**). Our additional experiment confirmed that *P. duboscqi* is not permissive to *L. infantum* development and can therefore be classified as a specific vector. Moreover, lectin affinity blotting revealed that unlike *P. perniciosus* and *P. argentipes*, there are no N-acetyl-D-galactosamine- displaying epitopes in *P. duboscqi* midgut (Fig 4). Such glycoconjugates have been suggested as potential *Leishmania* ligands in the midgut of permissive vector species [20]. In conclusion, this study gives supporting evidence to the present distinction of sand flies into categories based on their susceptibility to various *Leishmania* species [1]. Together with the results of Myskova *et al.* [20] and Rogers *et al.* [29], our studies of *L. major lpg1* ^−^ development suggest the presence of a an LPG-independent parasite-binding mechanism within the midgut of permissive sand flies.

Unlike LPG-deficient *lpg1* ^−^ mutants, PG-deficient *lpg2* ^−^ mutants additionally were impaired in early development in sand fly and unable to survive at all stages in all sand fly species tested. In *P. argentipes* and *P. duboscqi* parasites of this line are severely impaired as early as day 2 post infection. For *P. duboscqi*, these data stand in line with those of Boulanger *et al.* [32]. A similar finding was described for PG-deficient mutants of *L. donovani* in *P. argentipes* by Sacks *et al.* [4]. Moreover, Secundino *et al.* (submitted) have recently made similar observations in *P. duboscqi* originating from Mali and in *Lu. longipalpis* (see **Table 1**).

It has been hypothesized that parasite death in the pre-defecated sand fly midgut is attributable to digestive enzymes and that the phosphoglycans other than LPG confer resistance to the proteolytic attack [33], specifically phosphoglycans dependent on the activity of *LPG2*. While *LPG2* has been suggested to affect synthesis of other glycoconjugates beyond PGs, this possibility was excluded through studies of a second PG-deficient mutant, defective due to a lack of the UDP-Gal transporters *LPG5*A and *LPG5B* by gene targeting, showing that it is also unable to survive the late stages of *P. duboscqi* infection (Secundino *et al.*, submitted). Thus, it is likely that the defects in *lpg2* ^−^ infection of midguts described here and previously arise primarily through loss of PGs other than LPG, potentially the PPGs common to all species, or sAPs which occur in *L. major* albeit to lesser extents than in other *Leishmania* species [34].

In this work we also attempted to assess the importance of *LPG2*-dependent molecules by exposing parasites to the action of a proteolytic enzyme *in vitro*. As trypsin-like proteases were described as the most abundant digestive enzymes in both *P. papatasi* and *Lu. longipalpis* midgut after bloodfeeding [35],[36], bovine panceratic trypsin was chosen for these experiments. The bovine enzyme used shares all the conserved amino acid residues that influence the substrate specificity with sand fly midgut trypsin-like molecules. The *lpg2* ^−^ promastigotes lacking surface PGs did not prove to be more vulnerable to trypsin activity than the WT and *lpg1* ^−^ parasites whose resistance to trypsin has been previously reported [13]. These results could argue against a role of PGs in conferring resistance of promastigotes to the trypsin-like digestive enzymes in the sand fly gut. However, in light of the studies of Secundino *et al* (submitted), we think it more likely that bovine trypsin is not a good model for the activity and/or properties of all the proteolytic contents of the sand fly midgut. Most importantly, Secundino *et al* showed that inhibition of tryptic and other proteolytic activity in the midgut does in part rescue the survival of *lpg2* ^−^ parasites, although they do not exclude the possibility that also other factors contribute to the inability of the *lpg2* ^−^ to survive within the bloodmeal. Sand fly immunity has been shown to play a major role in the control of bacterial and parasitic infections [32] and potentially that molecules such as antimicrobial peptides secreted to the midgut lumen could contribute to the destruction of the *lpg2* ^−^ mutant parasite.

In summary, this study demonstrates that an LPG-independent mechanism of attachment of *Leishmania* is a feature common to permissive sand fly species. It also proves the importance of *LPG2*-dependent molecules in the survival of *L. major* in various sand fly vectors. Moreover, it brings an evidence that *P. duboscqi* is not able to support development of *L. infantum* and therefore can be classified as a specific vector.
